# Treating inborn errors of liver metabolism with stem cells: current clinical development

**DOI:** 10.1007/s10545-014-9691-x

**Published:** 2014-03-26

**Authors:** Etienne Marc Sokal

**Affiliations:** Pediatric Hepatology & Gastroenterology and Cell Transplant Center, Université Catholique de Louvain, Cliniques Universitaires Saint-Luc, 10 av Hippocrate, 1200 Brussels, Belgium

## Abstract

Advanced therapies including stem cells are currently a major biotechnological development. Adult liver stem cells can differentiate into hepatocyte like cells and be infused in the recipient’s liver to bring a missing metabolic function. These cells can be produced in large quantities in vitro. Allogeneic stem cells are required to treat genetic diseases, and this approach allows to use one single source of tissue to treat different diseases and many recipients. Mesenchymal stem cells can in addition play an immunomodulatory and anti-inflammatory role and possibly prevent the accumulation of fibrous tissue in the liver. From a regulatory point of view, stem cells are considered as medicinal products, and must undergo a pharmaceutical development that goes beyond the research and proof-of-concept phases. Here, we review the track followed from the first hepatocyte transplantation in 2000 to the next generation product issued from stem cell technology, and the start of EMA approved clinical trials to evaluate the safety and potency of liver stem cells for the treatment of inborn errors of the liver metabolism.

Liver regenerative medicine aims to repair a functional defect using cells, with metabolic diseases as the main indication (Smets et al 2008; Sokal 2011; Nussler et al 2006; Dhawan et al 2010).

Besides metabolic repair, stem cells are also able to restore parenchymal integrity and structure, for example by reducing fibrosis in chronic diseases (Forbes and Newsome 2012). Mesenchymal stem cells can also reverse inflammation in various toxic, autoimmune and alloimmune mediated liver injuries (rejection, graft-versus-host reactions) (Forbes and Newsome 2012; Le Blanc et al 2008; Sana et al 2013; Kean et al 2013).

These different targets are based on very different principles of action. In the first case, the principle is based on introducing in the deficient recipient liver healthy cells and their enzyme content (Smets et al 2008; Sokal 2011; Nussler et al 2006; Stéphenne et al 2005, 2006, 2012; Sokal et al 2003; Lysy et al 2008a). In the second case, the cells introduced act through cytokines and signaling factors as well as through their immunoregulatory properties, which can modify cellular interactions and re-establish a physiological balance between parenchyma, inflammatory cells, and stellate or myofibroblastic cells (Forbes and Newsome 2012; Sana et al 2013; Remberger et al 2008).

In metabolic diseases, cell grafts have now been developed for around 15 years, the first clinical case being a Crigler-Najjar patient treated with hepatocytes (Fox et al 1998). The liver cells are obtained by enzymatic dissociation of the liver using collagenase (Sokal et al 2003). Then, centrifugation separates the parenchymal fraction, which contains the hepatocytes (80 % of the total), from the non-parenchymal fraction, which contains, amongst others, stellate cells (8 %, precursors of myofibroblasts), macrophages known as Kupffer cells, epithelial duct cells and endothelial cells. When used for clinical purpose, the process of collagenase perfusion and cell isolation must be performed in approved tissue banks, working under the EU directive on tissues and organs. Whenever stem cells are produced and manipulated, the EU regulation on advanced therapies applies in addition to the EU directive on tissues and organs.

Hepatocyte grafts have shown that cellular therapy is technically feasible and have established the proof of concept that it is possible to correct deficient enzymatic activity in the recipient liver using cells.

The cells are delivered via a catheter placed in the portal vein of the recipient, either by the percutaneous route, either via an implantable catheter such as a Porth-a-Cath (Darwish et al 2004). Direct intra-parenchymal injection has also been tested successfully in pre clinical models, and the engraftment of the cells can furthermore be improved by embedding the cells in a matrix support such as hyaluronic acid or matrigel.

The percentage of liver cell replacement considered as necessary to significantly improve metabolic disorders is around 5 % of the total liver mass, while 10 % could normalize the function. Asonuma showed that transplanting an auxiliary liver corresponding to 12 % of the whole liver mass was as efficient as a whole organ to correct hyperbilirubinemia in rat (Asonuma et al 1992). In human, infusing 5 % of the estimated liver mass was sufficient to decrease significantly the bilirubin level in Crigler-Najjar patients. (Fox et al 1998; Lysy et al 2008b; Ribes-Koninckx et al 2012). In a mouse model of phenylketonuria, 4.8 % to 9.3 % repopulation with wild type hepatocytes allowed to decrease phenylalanine levels by 68 %, considered as an appropriate therapeutic goal in patients. Phenylalanine levels normalised in animals having 10 % liver repopulation (Harding and Gibson 2010). This figure was corroborated in a child who achieved a significant reduction of phenylalanine levels and half life after hepatocyte transplantation, with 13 % and 6.6 % of phenylalanine hydroxylase activities measured in the post transplant liver biopsies (Stéphenne et al 2012).

In a child with arginosuccinate lyase deficiency, significant metabolic and clinical improvement was obtained with infusion of 9 % of her liver mass, providing a 3 % de novo measured enzyme activity (Stéphenne et al 2006). As another example, living donor liver transplantation using a donor with 8 % of activity was able to correct the phenotype in a child with citrullinemia. (Ban et al 2001).

The figure usually proposed that 5 % of the liver mass is equivalent to 250 million cells per kg body weight is based on estimations (Fox et al 1998), and seems probably by far underestimated for small infants for who the classical doses of 200 million cells par kg body weight is probably much less than 5 % (Meyburg et al 2009a). The number of hepatocytes in the human liver was calculated to be 2.41 × 10^11^ (241 billion cells), accounting for 80 % of the total liver cell mass. Stellate cells were calculated as 10 % of the hepatocyte mass; 5 % of the hepatocyte mass would then be 12 billion cells for an adult (Bianconi et al 2013). Most of the hepatocyte transplantation programs deliver 2 to 4 billion cells to the recipient liver. Not all transplanted cells engraft, as the cells are trapped in the portal sinusoids and may be lost before crossing the endothelial barrier. Drugs such as NSAIDs, nitroglycerin or prostacyclin (Bahde et al 2013) or radiation therapy are being tested to facilitate this limiting step by opening the endothelial wall.

Hepatocytes, but also liver stem cells are poorly immunogenic and even tolerigenic; they do weekly express HLA class I antigens and FAS, but not HLA-DR, nor FAS ligand nor co-stimulatory molecules. In co-culture with allogeneic T cells, they do not induce T cell activation nor proliferation, and hepatocytes induce IL 10 production by dendritic myeloid cells inducing naïve CD4+ T cell hypo-responsiveness (Sana et al 2013). However, no precise clinical correlation allows at this stage to withdraw immunosuppression in allogeneic liver cell transplantation.

Given the limited percentage of cells delivered to patients, it is therefore quite remarkable that functional improvements have indeed been observed in various severe metabolic conditions. The best results have been obtained in urea cycle disorders (Stéphenne et al 2005, 2006; Meyburg et al 2009b; Pareja et al 2013), in Crigler–Najjar syndrome (Lysy et al 2008a; Fox et al 1998; Pareja et al 2013; Ambrosino et al 2005), infantile Refsum’s disease (Sokal et al 2003), factor VII deficiencies (Dhawan et al 2004), phenylketonuria (Stéphenne et al 2012), glycogen storage disease type I (Pareja et al 2013; Muraca et al 2002), Refsum disease (Sokal et al 2003) and tyrosinemia (Pareja et al 2013). More mitigated results have been observed in hypercholesterolemia and organic acidemias.

The results of published cases of hepatocyte transplantation have been recently reviewed (Jorns et al 2012). The main limitation is that the duration of the clinical effect is limited, and the benefit is lost after a period that varies between a few months and a little more than 1 year, the maximum being 18 months. This observation means that the method is, at its current stage, not attractive enough for patients and metabolic doctors.

These case reports have been performed as proof of concept cases and not within proper clinical trials. This innovative medical practice is of course important at the emergence of innovative therapies; however, only successful cases are published, as physicians and medical journals hardly report/accept case reports, which do not show efficacy. It is therefore hard to draw firm conclusions about safety and efficacy of the procedure, and about the percentage of children who do effectively benefit. In addition, adverse events are not systematically collected for all cases performed and are not reported as it would be in clinical trials; efficacy end points are in addition not firmly established in advance, and the protocol is often modified. This illustrates the richness and limitations of such medical innovations, and the necessity to proceed to clinical trials. For hepatocytes, Cytonet (Weinheim, Germany) is currently conducting such trial in urea cycle disorders, while Promethera Biosciences (Mont-St-Guibert, Belgium) is conducting trials with liver stem/progenitor cells in urea cycle disorders and Crigler-Najjar syndrome.

Amongst the main adverse events to consider are the risk of infectious agent transmission by fresh or even cryopreserved liver cells, which are comparable to agents transmitted by whole liver graft (Bacteria, Epstein Barr virus, Cytomegalovirus, ect.). Hence, hepatocyte transplantation faces the same risk of causing post transplant lymphoproliferative diseases related to Epstein Barr virus.

In hepatocyte transplantation, each transplant procedure requires more or less one source liver. Scarcity of donors is not compatible with universal availability of this procedure, which remains limited for a few patients in selected transplant centers. Excess hepatocytes are commonly cryopreserved, but these highly metabolically active cells are damaged by the cryopreservation process, which causes mitochondrial respiratory chain and membrane permeability damages (Stéphenne et al 2007).

Liver stem/progenitor cells are obtained form primary hepatocyte cultures. Plating the liver cells contained in the parenchymal fraction for primary culture leads to the emergence of different subpopulations of progenitor cells, which can expand in culture and retain an ability to differentiate into hepatocytes (Najimi et al 2007). Other candidate stem cell types have been isolated from the liver using different culture systems (Herrera et al 2006). This technology is the source of a great deal of hope – producing cells in vitro on an almost unlimited scale that can correct metabolic defects.

This in vitro expansion makes it possible to break away from the dependency on organ donation, as the quantity of source tissue necessary becomes negligible. In addition, this is particularly compatible with a high scale pharmaceutical production, and this gives a real prospect to bring cell-based therapy to any patient in need in any metabolic centre. Pharmaceutical development is also the guarantee to conduct proper clinical trials, evaluating both safety and efficacy. Stem or progenitor cells, produced in vitro in conditions of good manufacturing practice, are classed as medicinal products in Europe. They are known as advanced therapy medicinal products (ATMPs), and this status is defined by a specific European framework (http://www.ema.europa.eu/ema/index.jsp?curl=pages/regulation/general/general_content_000294.jsp and a regulation issued in 2007 (n°1394/2007)

http://ec.europa.eu/health/files/eudralex/vol-1/reg_2007_1394/reg_2007_1394_en.pdf. A specific committee has been created at the European Medical Agency, the Committee for Advanced Therapies (CAT) which is in charge of following clinical development of all cell therapy products. Cell based medicinal products can also be classified as orphan drugs if the target is a disease with an incidence lower than 1/5000 live births. For children, a Pediatric Investigation Plan must also be designed and submitted for evaluation by the Pediatric Committee. The European Medicinal Agency also offers scientific advices and protocol assistance.

The european regulation authorizes hospital accredited tissue banks to treat a few patients with ATMPs under the so called “hospital exemption” rule. This is most important to preserve, as it allows researchers and physicians to explore new targets for cell therapies. Once the “invention” and proof-of-concept phases have been passed, the clinical development can only be considered at a pharmaceutical level and must follow all regulatory steps for medicinal product market authorization.

Thus was the implantation and repopulation of a recipient liver by liver stem cells produced in vitro first shown in a patient suffering from a urea cycle defect (Sokal et al 2013). In this patient with a severe form of ornithine carbamoyl transferase deficiency, the recipient liver repopulation with donor cells reached 3 to 5 %. This meant that infused cells had proliferated, as the patient had received around 0.75 % % of her liver mass. Such in vivo repopulation with the Adult Derived Human Liver Stem Cells (ADHLSC) was previously shown in uPA SCID mice and SCID mice, with differentiation into hepatocyte like cells producing human protein and enzymes (Najimi et al 2007; Khuu et al 2013). When indium labelled cells were infused in the portal vein in a patient with type I glycogen storage disease, exclusive liver homing was demonstrated up to the fifth day after infusion (Defresne et al 2014).

Development of ATMP must follow a strict production plan, in an approved Good Manufactory Practice (GMP) pharmaceutical environment. Release criteria must be established and approved, including among other specification the percentage of viability, the stability, level of impurities, precise cell identification markers, genetic stability and potency to treat the target disease. The process must also be adapted according to logistical requirements to deliver the cell therapy medication to the patient’s bedside, which can be far from the production site. The ideal scope will be to store the product in the hospital pharmacy and perform the reconstitution of the drug substance at the patient’s bed, as currently done for vaccines.

The potency is the cell metabolic capacity that matches the disease targeted: production of urea, bilirubin conjugation, glucose synthesis, production of coagulation factors, and so on. Such potencies have been confirmed for adult-derived human liver stem cells (ADHLSCs) (Najimi et al 2007; Khuu et al 2011). Preclinical tests have also shown their in vivo differentiation after transplantation in mice (Najimi et al 2007; Khuu et al 2013).

Preclinical studies must evaluate the safety, absence of oncogenicity, absence of toxicity, and biodistribution of the cells (Scheers et al 2012). Before the end of the clinical development, animal safety and biodistribution studies have to be repeated in Good Laboratory Procedure (GLP) environment.

Clinical studies must adhere to a development plan approved by European and national health authorities. The first phase I/II studies aim to demonstrate safety and to gauge the optimal dose of cells that needs to be infused (dose-finding studies). A second phase will serve to confirm dose and efficacy. Long term safety follow up of the patients must be organized and any adverse event collected and reported. As a last step, the pivotal study, will aim to confirm efficacy with a finalized production procedure and will enable to apply for marketing authorization for the medication, delivered ultimately by the European commission.

In the case of adult derived human liver stem cells, the pharmaceutical development is ongoing; 20 patients have been included in the phase I/II study, including Crigler-Najjar and urea cycle disorders. Patients have been treated in France, the UK, Italy, Belgium and Israel. This development is performed by Promethera Biosciences (Mont-St-Guibert, Belgium), a spinoff company of the Université Catholique de Louvain.

The challenge, of course, will be to ascertain whether the quantities of cells infused, which are theoretically around 1–2 % of the mass of the recipient’s liver, will deliver a metabolic benefit that is quantitatively sufficient. The mechanism also lies in their capacity to survive on a long-term basis and to proliferate, thus increasing metabolic efficacy over time.

As discussed above, in many metabolic diseases, a small percentage of enzyme activity (a few per cent) can considerably modify the phenotype of a patient, transforming a severe disease that is difficult to control into a more favourable phenotype, with a significant improvement in the quality of life of the patient and the patient’s family.

For ADHLSC, the product is to be thought of as a treatment that will be part of the therapeutic arsenal available to metabolic doctors. Its function will be to improve the metabolic balance of unstable patients for whom a liver transplant is not planned. Although liver transplantation is a more radical technique for correcting a metabolic defect, it is not without risk, both in the short and long term. In a large retrospective study of 323 children transplanted for urea cycle and organic acid disorders, small children less than two years of age were at increased risk of graft loss (78 % graft survival at 5 years), whilst the 5 years graft survival in older kids was 88 %. Eight children had died on the waiting list before receiving an organ (Perito et al 2014). Long term graft survival can be hampered by de novo immune hepatitis and chronic fibrosis of the graft (Evans et al 2006; Andries et al 2001), while chronic or acute rejection in adolescence can lead to re-transplantations as an adult. The current shortage of organs creates difficulties to define priorities (Donckier et al 2013). The survival rate of paediatric liver transplantation averages 85 to 95 % at one year, decreasing to 75–85 % in the longer term (Wallot et al 2002; Goss et al 1998; Bourdeaux et al 2009).

Stem cell based technology is a new biotechnology approach to treat patients with liver based metabolic diseases. The proof of concept is made, and clinical trials are on-going to design the best approach to deliver a sufficient quantity of cells to change the clinical phenotype of the patients. Preliminary data, including first in man treatments and on-going clinical studies bring the expected evidence that this therapy will break through in the coming years. Proper clinical trials have now to emerge in continuity with single case studies.
